# Kinematic Alignment in Total Knee Arthroplasty of Varus Knees Minimises Distal Ankle Compensatory Changes Compared with Mechanical Alignment

**DOI:** 10.3390/jcm15124687

**Published:** 2026-06-17

**Authors:** Joaquín Moya-Angeler, Pablo Sánchez-Urgelles, Carmelo Marín-Martínez, Simon Nurettin van Laarhoven, Matteo Innocenti, Mustafa Akkaya, Filippo Leggieri, Antonio Klasan, Francisco Lajara-Marco, Vicente J. León-Muñoz

**Affiliations:** 1 Olympia Medical Center, 28046 Madrid, Spain; jmoyaangeler@gmail.com; 2 Instituto de Cirugía Avanzada de la Rodilla (ICAR), 30005 Murcia, Spain; vleonmd@gmail.com; 3 Independent Researcher, 28691 Madrid, Spain; sanchezurgelles.pablo@gmail.com; 4 Department of Orthopaedic Surgery and Traumatology, Hospital General Universitario Reina Sofía, 30003 Murcia, Spain; drlajaramarco@gmail.com; 5 Department of Orthopaedic Surgery, Sint Maartenskliniek, P.O. Box 9011, 6500 GM Nijmegen, The Netherlands; s.vanlaarhoven@maartenskliniek.nl; 6 Azienda Ospedaliero Universitaria Careggi, University of Florence, 50134 Florence, Italy; matteo.innocenti@unifi.it (M.I.); filippoleggieri@icloud.com (F.L.); 7 Department of Orthopaedics, Ankara Koru Hospital, 06510 Ankara, Turkey; mustafa@drakkaya.com; 8 Department of Orthopaedics, Yuksek Ihtisas University, 06520 Ankara, Turkey; 9 AUVA UKH Steiermark, 8020 Graz, Austria; klasan.antonio@me.com; 10 Department of Orthopedics and Traumatology, Johannes Kepler University Linz, 4040 Linz, Austria; 11 Instituto Murciano de Investigación Biosanitaria Pascual Parrilla (IMIB), 30120 Murcia, Spain; 12 Department of Surgery, Paediatrics and Obstetrics & Gynaecology, Faculty of Medicine, University of Murcia, 30120 Murcia, Spain

**Keywords:** total knee arthroplasty, kinematic alignment, mechanical alignment, ankle biomechanics, joint line obliquity

## Abstract

**Background/Objectives:** Alignment philosophy in total knee arthroplasty (TKA) may affect joints beyond the knee. Mechanical alignment (MA) targets a neutral mechanical axis, whereas kinematic alignment (KA) aims to restore native alignment and joint line obliquity (JLO). This study compares the effects of MA and KA on hip and ankle radiographic parameters and investigates the propagation of coronal correction along the lower limb. **Methods:** A retrospective comparative study evaluated 63 TKAs performed for varus deformity (KA: *n* = 32; MA: *n* = 31). Pre- and postoperative full-length standing radiographs were used to calculate changes (Δ), defined as the difference between postoperative and preoperative values, in hip offsets, mechanical and arithmetic hip–knee–ankle angles (mHKA, aHKA), medial proximal tibial angle (MPTA), lateral distal femoral angle (LDFA), JLO, and ankle ground-referenced angles. Between-group differences and correlations were analysed. Interobserver reliability was assessed for all variables. **Results:** MA produced significantly greater limb correction than KA (ΔmHKA: 8.89° vs. 4.82°, *p* < 0.001), primarily due to increased tibial valgus correction (ΔMPTA: 6.26° vs. 2.41°, *p* < 0.001). JLO increased substantially with MA (+4.10°) but was preserved with KA (+0.30°, *p* < 0.001). MA resulted in significant valgus shifts at the ankle (ground talar dome angle (GTDA) −3.01°, ground tibial plafond angle (GTPA) −3.02°; *p* = 0.006 for both), whereas KA produced no significant ankle changes. Correlation analysis demonstrated limited knee–ankle biomechanical coupling, with a moderate negative correlation in MA (ΔmHKA vs. ΔGTDA: ρ = −0.479, *p* = 0.006) and a weak correlation in KA (ΔaHKA vs. ΔGTDA: ρ = −0.360, *p* = 0.043). Hip parameters remained unchanged in both groups. **Conclusions:** Mechanical alignment induces larger tibial-driven coronal corrections, increases joint line obliquity, and produces measurable valgus shift at the ankle. In contrast, kinematic alignment preserves native alignment and joint-line obliquity while minimising distal ankle compensatory changes.

## 1. Introduction

Restoring coronal alignment remains a central principle of total knee arthroplasty (TKA). Mechanical alignment (MA) is traditionally achieved by orienting bone cuts perpendicular to mechanical axes to produce a neutral limb [1]. In contrast, kinematic alignment (KA) seeks to reproduce the patient’s pre-arthritic alignment by restoring native joint lines and soft-tissue balance [2].

While functional outcomes and implant longevity have been studied for both MA and KA, the specific impact of alignment strategy on adjacent joints above and below the knee remains less well understood [3,4,5]. The hip’s ball and socket configuration allows substantial coronal plane adaptability, whereas the ankle mortise is inherently constrained, potentially rendering it more susceptible to stress transfer following knee realignment. Previous studies have shown that correction of varus deformity with TKA can induce compensatory changes at the ankle and hindfoot, particularly in patients with limited subtalar mobility, potentially altering load distribution or causing symptoms [6,7,8,9,10,11,12]. However, the mechanisms underlying these adaptations, specifically whether tibial or femoral-driven corrections predominantly drive distal compensation, remain unclear. Varus knee deformity is known to be predominantly tibial in origin in most osteoarthritic knees, with the medial proximal tibia contributing more to overall coronal malalignment than the distal femur, suggesting that tibial correction may play a greater role than femoral correction in driving overall limb realignment and distal compensatory changes. In addition, it is uncertain whether the alignment philosophy chosen during TKA influences hip radiographic parameters [13,14].

Recent evidence has increasingly supported the concept that knee realignment after TKA may propagate distally. Portes-Chiva et al. reported that mechanical-axis correction after TKA produced significant changes in ankle joint orientation and that the magnitude of knee-axis correction correlated with radiographic ankle adaptation, although these changes were not associated with worse ankle-related patient-reported outcomes at 1 year. Similarly, Spadini et al., in a large cohort of 4698 mechanically aligned TKAs, showed that both preoperative and postoperative HKA alignment were associated with ground-referenced tibial plafond and talar dome orientation, whereas talar tilt remained largely unaffected. These findings suggest that postoperative ankle changes may reflect a coordinated reorientation of the distal tibia–talus complex relative to the ground rather than isolated intra-articular ankle deformation. However, comparative evidence directly evaluating whether KA and MA produce different hip–knee–ankle propagation patterns remains limited.

Joint line obliquity (JLO) may be an important element in this interaction between knee realignment and distal adaptation. By preserving native joint line obliquity, KA may maintain a coronal orientation closer to the patient’s constitutional anatomy. Conversely, MA frequently requires a greater modification of tibial orientation to achieve a neutral mechanical axis, which may increase joint line obliquity and alter load transmission along the kinetic chain [15,16]. This difference provides a potential biomechanical explanation for why distal ground-referenced ankle parameters may respond differently after MA and KA.

Despite increasing interest in personalised alignment strategies, several questions remain unresolved. First, it is unclear whether the choice of alignment philosophy itself influences the propagation of coronal correction beyond the knee. Second, the relative contribution of tibial versus femoral correction to distal ankle radiographic changes remains insufficiently defined. Third, it remains uncertain whether preservation of joint line obliquity with KA is associated with fewer compensatory radiographic changes at the ankle.

Therefore, this study examined whether different alignment philosophies in TKA resulted in distinct radiographic alignment effects at the hip, knee, and ankle in varus knees. By comparing KA and MA, the research aimed to determine whether the rationale behind KA (restoring native alignment and preserving JLO) resulted in fewer compensatory changes at the ankle and better maintenance of proximal radiographic alignment than MA, which focused on achieving a neutral mechanical axis. We hypothesised that (1) MA would produce greater ankle alignment changes than KA due to more substantial realignment, (2) overall limb correction would correlate more strongly with tibial than femoral correction, and (3) KA would better preserve JLO and proximal radiographic alignment, clarifying whether alignment philosophy should be considered to minimise distal joint stress.

## 2. Materials and Methods

### 2.1. Study Design

A retrospective comparative study of consecutive primary total knee arthroplasties with varus alignment (mHKA < 177°) was performed, including only varus knees in order to maximise internal validity, avoid measurement heterogeneity, and allow comparability with previous studies [17,18]. Case selection was based on institutional and temporal availability: MA TKAs were performed at HLVLG between 2017 and 2018, whereas KA TKAs were performed at HCUVA between 2019 and 2020 (including the COVID-19 pandemic period) and at HGURS between 2021 and 2022, covering an overall inclusion period from 2017 to 2022. This temporal allocation reflects the surgeon’s transition in alignment philosophy: MA procedures performed before the adoption of KA, and kinematically aligned procedures performed thereafter, accounting for the broader surgical time frame from 2017 to 2022.

Exclusion criteria included prior ipsilateral hip or ankle arthroplasty, extra-articular deformity, inflammatory arthritis, or symptomatic hip or ankle pathology.

A total of 63 knees met criteria (KA: *n* = 32; MA: *n* = 31). Sample size was determined a priori for inter- and intraobserver reliability analysis. Assuming two observers, an expected intraclass correlation coefficient (ICC) of 0.80, and a 95% confidence interval width of ±0.10 for key hip and ankle parameters, a minimum of 31 knees was required. The final cohort of 63 varus knees, therefore, provided adequate precision for reliability assessment.

Based on the final sample (KA: *n* = 32; MA: *n* = 31), post hoc power analysis (two-sided α = 0.05) using observed effect sizes (Cohen’s d) demonstrated high power for the main outcomes, including Δ values defined as the difference between postoperative and preoperative measurements for ΔmHKA (98%), ΔMPTA (99%), ΔJLO (>99%), ΔGTDA (95%), and ΔGTPA (96%). Power was low (≈20%) for ΔLDFA, consistent with its non-significant between-group difference.

### 2.2. Surgical Technique

**Figure 1:**
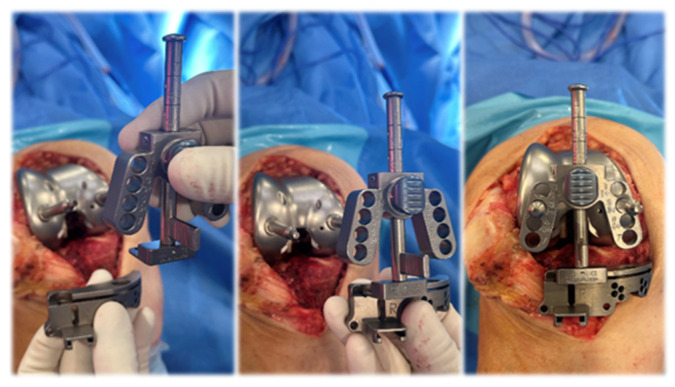
Components and assembly of the link device system used during kinematic alignment. (**Left**): femoral trial with two alignment rods, link device, and floating tibial jig. (**Middle**): connection of the link device to the tibial jig. (**Right**): fully assembled construct attached to the femoral trial with the knee positioned at ninety degrees of flexion.

All operations were performed by a single surgeon (VLM) experienced in both techniques. All patients received a cemented medial pivot total knee prosthesis (GMK Sphere, Medacta International, Castel San Pietro, Switzerland). In the MA group, tibial and femoral bone cuts were oriented perpendicular to the mechanical axes to achieve neutral alignment, and soft tissue releases were performed as required. In the KA group, a calliper-measured resection technique was used to restore the native distal and posterior femoral joint lines, and tibial resection was determined using a linked femur to tibia device and a floating tibial jig, guided by native medial and lateral compartment tension with the knee positioned at 90 degrees of flexion (Figure 1) [19,20].

### 2.3. Imaging and Measurements

Standardised full-length standing radiographs were obtained pre- and postoperatively. Standardisation included a fixed source-to-image distance, with patients standing upright, hips and knees fully extended, feet positioned 10 cm apart, and the patellae oriented forward and centred over the femoral condyles.

**Figure 2:**
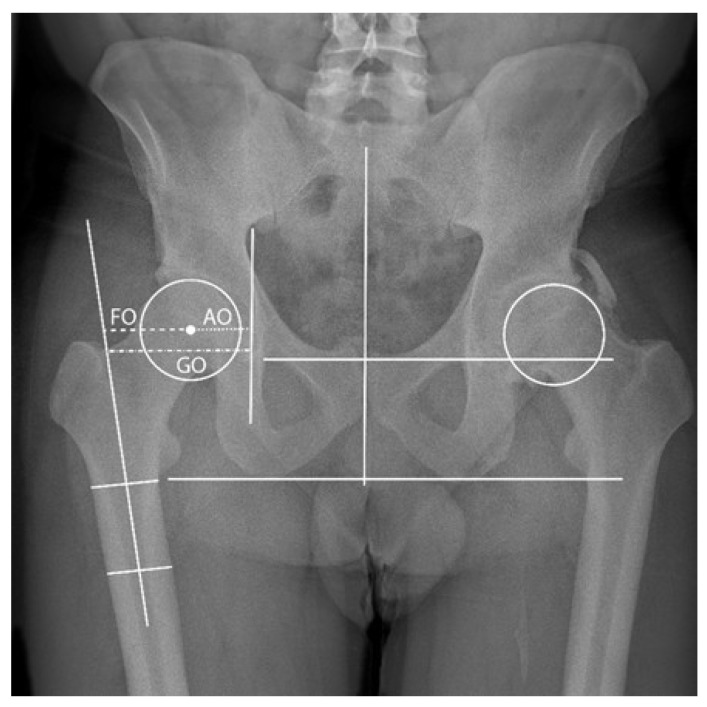
Hip measurement parameters, including femoral offset (FO), acetabular offset (AO), and global offset (GO).

**Figure 3:**
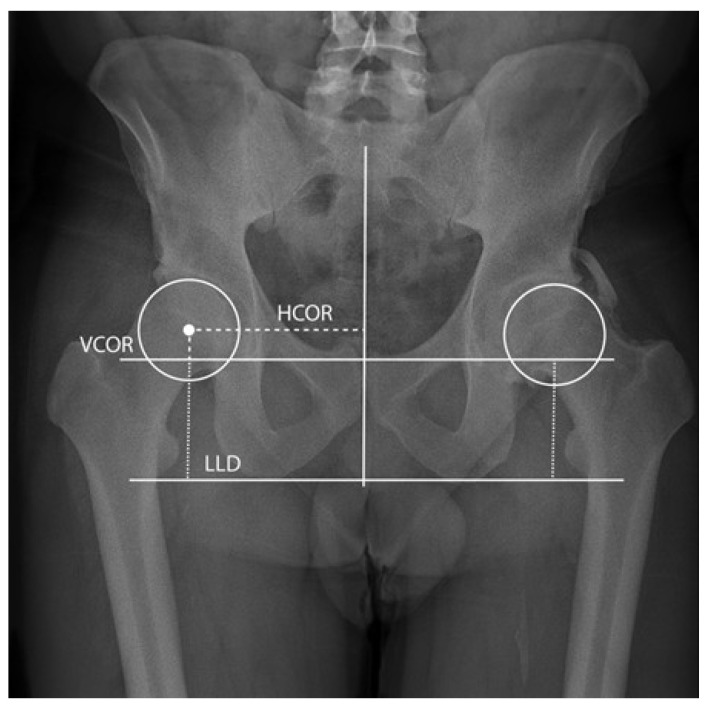
Hip measurement parameters, including vertical centre of rotation (VCOR), horizontal centre of rotation (HCOR), and limb length discrepancy (LLD).

Hip parameters included femoral offset (FO), acetabular offset (AO), global offset (GO), vertical and horizontal centre of rotation (VCOR, HCOR), and limb length discrepancy (LLD) (Figure 2 and Figure 3).

Knee parameters included mechanical hip knee ankle angle (mHKA), arithmetic hip knee ankle angle (aHKA), medial proximal tibial angle (MPTA), lateral distal femoral angle (LDFA), knee joint line convergence angle (KJLCA), and joint line obliquity (JLO).

**Figure 4:**
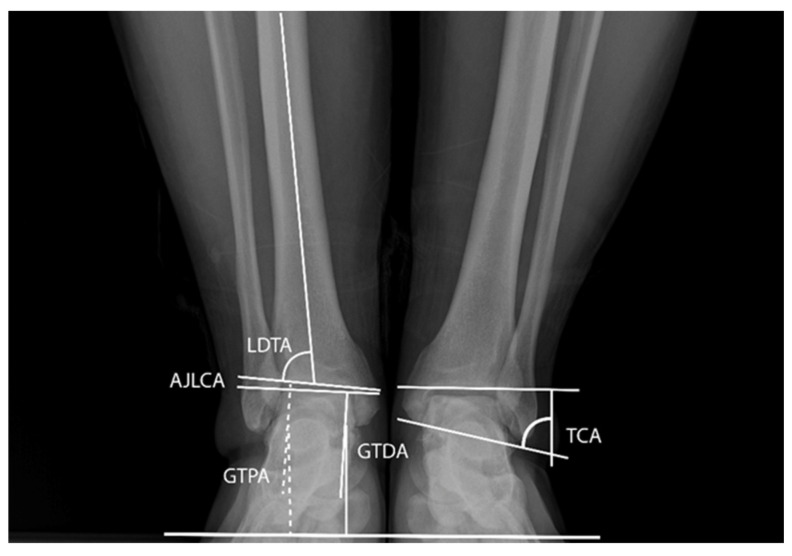
Ankle measurement parameters, including lateral distal tibial angle (LDTA), ankle joint line convergence angle (AJLCA), ground talar dome angle (GTDA), ground tibial plafond angle (GTPA), and talocrural angle (TCA).

Ankle parameters included both ground-referenced and intra-articular measurements. The ground talar dome angle (GTDA) was defined as the orientation of the talar dome relative to the ground, and the ground tibial plafond angle (GTPA) as the orientation of the tibial plafond relative to the ground. These parameters were used to evaluate distal ankle orientation in relation to the weight-bearing axis. The lateral distal tibial angle (LDTA) described coronal distal tibial alignment, the talocrural angle (TCA) reflected the relationship between the distal tibia and talus, and the ankle joint line convergence angle (AJLCA) assessed convergence between the tibial plafond and talar dome. This distinction allowed differentiation between ground-referenced ankle reorientation and intra-articular ankle alignment changes (Figure 4).

All measurements were performed independently by two evaluators using the institutional PACS software Syngo Share VA28B version 4.8.0.1011 (Siemens Healthineers, Erlangen, Germany). Intraclass correlation coefficients (ICCs) were calculated to assess inter-rater reliability using absolute agreement. ICC values > 0.75 were considered excellent, 0.60–0.74 good, 0.40–0.59 fair, and <0.40 poor.

### 2.4. Measurement Reliability

Interobserver reliability analysis demonstrated robust measurement reproducibility across all assessed hip, knee, and ankle radiographic variables, with intraclass correlation coefficients ranging from 0.73 to 0.95, indicating good to excellent agreement.

### 2.5. Study Endpoints

The primary outcomes were between-group and within-group differences in pre- to postoperative changes (Δ) of hip, knee, and ankle alignment parameters.

The secondary outcomes included correlations between knee correction magnitude (ΔmHKA, ΔMPTA, ΔLDFA) and ankle alignment changes (ΔGTDA, ΔGTPA, ΔLDTA), evaluation of tibial versus femoral contribution to overall limb realignment, and assessment of JLO preservation.

### 2.6. Data Analyses

Data normality was assessed using the Shapiro–Wilk test, which is appropriate for sample sizes below 50 (MA: *n* = 31, KA: *n* = 32). Most angular measurements demonstrated normal distribution and are presented as mean ± standard deviation. Variables showing significant deviation from normality (*p* < 0.05) are presented as median (interquartile range). 

Specifically, ΔLDFA and ΔaHKA in the KA group, ΔJLO in the MA group, ΔGTDA, ΔTCA, and ΔAJLCA in the MA group, and pre- and postoperative AJLCA values.

Baseline characteristics were compared between groups using independent *t*-tests for normally distributed variables, Mann–Whitney U tests for non-normally distributed variables, and chi-square tests for categorical variables.

Within-group pre- to postoperative changes were assessed using paired *t*-tests or Wilcoxon signed-rank tests based on distribution. Between-group differences in change scores (Δ values) were compared using independent *t*-tests for normally distributed variables or Mann–Whitney U tests for non-normally distributed variables.

Correlations between knee correction parameters and ankle alignment changes were assessed using Pearson correlation coefficients for normally distributed variable pairs and Spearman rank correlation coefficients when at least one variable was non-normally distributed. Specifically, Spearman correlations were used for all pairs involving ΔGTDA (MA group), ΔaHKA (KA group), ΔJLO (MA group), and ΔLDFA (KA group). Inter-observer reliability was evaluated using the intraclass correlation coefficients (ICC) with a two-way mixed-effects model.

Statistical significance was set at *p* < 0.05. All analyses were performed using R version 4.3.0 (R Core Team, 2023. R: A Language and Environment for Statistical Computing. R Foundation for Statistical Computing, Vienna, Austria. https://www.R-project.org/).

## 3. Results

A total of 63 knees (63 patients) met the inclusion criteria and were included in the final analysis. There were no missing data for primary outcome variables.

### 3.1. Demographics and Baseline Characteristics

The groups were well matched for age, sex, BMI, and baseline alignment (Table 1 and Table 2). Preoperative hip, knee, and ankle measurements showed no significant differences between groups, confirming comparable baseline deformity.

### 3.2. Knee Alignment Changes

MA produced significantly larger coronal corrections compared to KA across all mechanical and arithmetic axes (Table 3). The magnitude of correction was predominantly driven by tibial valgus correction, with MA demonstrating a 159.8% greater tibial correction than KA. In contrast, femoral corrections were comparable between techniques, with only a small, non-significant difference favouring MA.

Joint line obliquity (JLO) increased markedly with MA but was effectively preserved with KA, demonstrating a 126.7% greater change with MA. This finding confirms that MA substantially alters native joint line orientation, whereas KA maintains pre-arthritic joint line obliquity. Knee joint line convergence angle (KJLCA) changes did not differ between groups.

### 3.3. Ankle Alignment Changes

MA generated significant valgus shifts in ground-referenced ankle angles, whereas KA produced no measurable changes (Table 4). Both GTDA and GTPA demonstrated statistically significant valgus angulation within the MA group, with large effect sizes (Cohen’s d > 0.9). The KA group showed no significant within-group changes for any ankle parameter.

LDTA trended toward greater valgus shift with MA compared to KA, approaching but not reaching statistical significance, with a medium effect size. Talocrural angle (TCA) and ankle joint line convergence angle (AJLCA) remained stable in both groups, suggesting that compensatory changes occur predominantly at the ground-referenced interface rather than within the mortise joint itself.

### 3.4. Knee–Ankle Coupling

**Figure 5:**
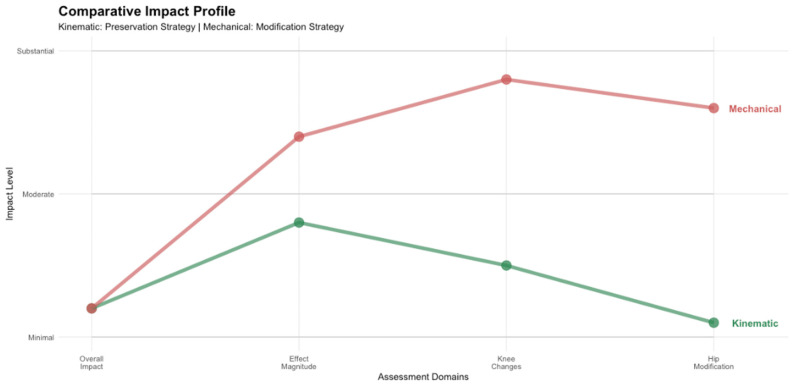
Comparative impact profile of mechanical (red line) and kinematic alignment (green line) across key assessment domains. Impact levels are displayed on a qualitative scale from minimal to substantial. Each point represents the relative impact magnitude within a specific assessment domain, and the points are connected solely to facilitate visual comparison of overall impact patterns between alignment strategies, without implying continuity or a quantitative relationship between domains. Mechanical alignment demonstrates greater overall impact, larger correction magnitude, and more substantial knee and distal joint modifications, whereas kinematic alignment consistently shows minimal alterations and preservation of native biomechanics.

**Figure 6:**
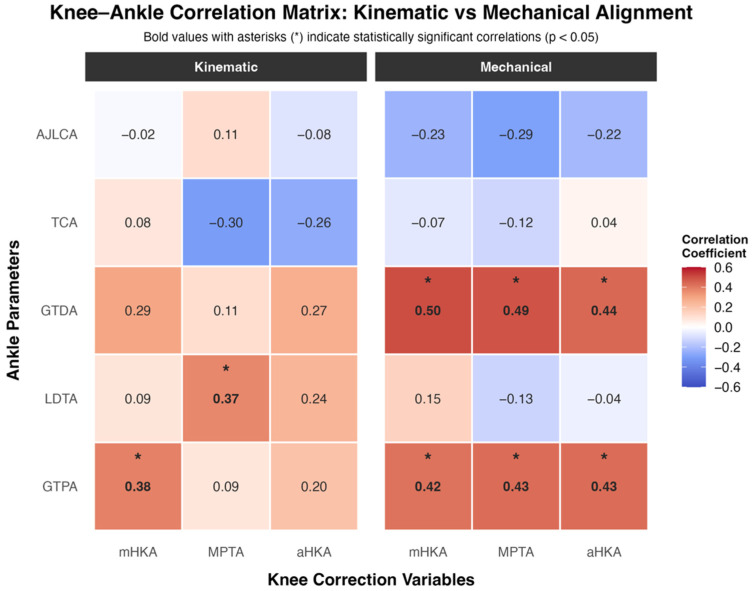
Correlation matrix between knee and ankle parameters in kinematic and mechanical alignment groups. The matrix shows correlation coefficients between knee correction variables, including mechanical hip–knee–ankle angle (mHKA), medial proximal tibial angle (MPTA) and arithmetic hip–knee–ankle angle (aHKA), and ankle parameters, including ankle joint line convergence angle (AJLCA), talocrural angle (TCA), ground talar dome angle (GTDA), lateral distal tibial angle (LDTA), and ground tibial plafond angle (GTPA). Mechanical alignment demonstrates stronger and more frequent correlations, indicating greater knee–ankle biomechanical coupling, whereas kinematic alignment shows minimal associations. Asterisks and bold indicate statistically significant correlations at *p* < 0.05.

**Figure 7:**
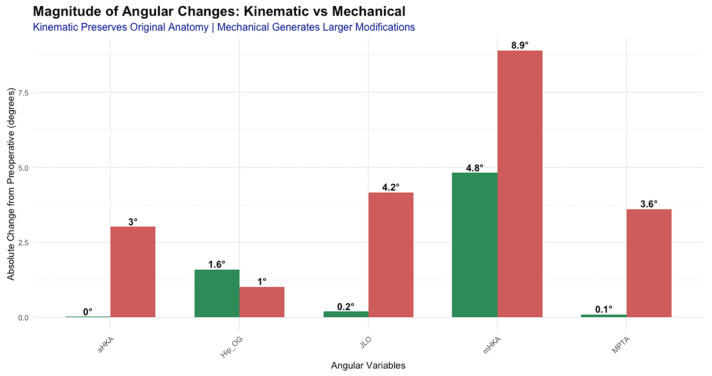
Absolute changes in key coronal alignment parameters comparing kinematic (green) and mechanical alignment (red). Bars illustrate postoperative modifications in the arithmetic hip–knee–ankle (aHKA), hip global offset (Hip_OG), joint line obliquity (JLO), mechanical hip–knee–ankle (mHKA) and medial proximal tibial angle (MPTA). Mechanical alignment produced larger angular corrections across all variables, whereas kinematic alignment preserved values closer to the native anatomy.

Correlation analysis revealed markedly different patterns of biomechanical coupling between groups (Figure 5, Figure 6 and Figure 7). The MA group demonstrated multiple significant correlations between knee correction magnitude and ankle parameter changes, with the strongest coupling observed between overall limb correction (ΔmHKA) and ground-referenced ankle angles (ΔGTDA, ΔGTPA). Moderate correlations were identified between tibial correction (ΔMPTA) and ankle valgus shifts, as well as between JLO changes and ankle adaptations.

In contrast, the KA group demonstrated minimal knee–ankle coupling, with only weak, isolated correlations between femoral and tibial parameters (ΔLDFA, ΔKJLCA) and specific ankle angles. Critically, no significant correlations were observed between overall limb correction (ΔmHKA, ΔaHKA) and any ankle parameter in the KA group, indicating biomechanical independence of the ankle joint following KA.

### 3.5. Hip Parameters

No significant changes in any hip parameter occurred with either alignment strategy. All measured offsets (FO, AO, GO), centres of rotation (VCOR, HCOR), and lower-limb length discrepancy (LLD) remained stable from pre- to postoperative assessments in both groups. These findings indicate that coronal knee realignment, regardless of alignment philosophy, does not influence proximal hip biomechanics or radiographic parameters.

## 4. Discussion

The most important finding of the present study is that alignment philosophy in TKA significantly influences the propagation of coronal correction to adjacent joints in varus knees. MA produced greater tibial-driven valgus shift, increased JLO, and induced measurable valgus shifts in ankle angles. In contrast, KA preserved JLO and did not produce significant ankle adaptations.

A possible explanation for these findings is the different way in which each alignment strategy modifies the coronal geometry of the limb. In varus knees treated with MA, correction toward a neutral mechanical axis usually requires a larger tibial valgus correction. This modifies the orientation of the proximal tibia and may secondarily change the relationship between the tibial plafond, talar dome, and the ground. This mechanism may explain why the most evident ankle changes were observed in ground-referenced parameters such as GTDA and GTPA, rather than in intra-articular ankle measures.

Conversely, KA aims to preserve constitutional alignment and native joint line orientation. By avoiding larger tibial-driven correction and maintaining joint line obliquity closer to the preoperative state, KA may reduce the magnitude of radiographic adaptation transmitted to the distal limb. These findings should be interpreted as radiographic and biomechanically plausible changes, not as direct evidence of clinical superiority, clinical harm, or symptomatic ankle benefit.

These findings suggest that JLO is associated with distal alignment changes, with greater increases in JLO observed in MA and minimal changes in KA. Preservation of JLO with KA was accompanied by fewer radiographic ankle alignment changes, without implying a direct causal effect. The moderate negative correlation between knee correction and ankle parameters in the MA group (ΔmHKA vs. ΔGTDA: ρ = −0.479, *p* = 0.006) suggests measurable but limited biomechanical coupling, with valgus knee correction associating with compensatory ankle varus. The weak correlation observed in KA (ΔaHKA vs. ΔGTDA: ρ = −0.360, *p* = 0.043) and the absence of other significant correlations suggest a more localised biomechanical effect. Notably, the inverse relationship indicates that the ankle compensates in the opposite direction to knee correction, potentially to maintain overall limb balance.

The study findings supported the proposed hypotheses. MA induced greater ankle changes than KA, as evidenced by a significant valgus shift. Tibial correction was found to influence the overall limb realignment more than femoral correction. KA preserved native biomechanics more effectively than MA, as evidenced by maintained JLO and the absence of ankle coupling.

Our results align with previous work showing hindfoot adaptations following varus correction using MA and extended those observations by including pure KA [6,8,9,10,11,12,21]. However, the limited number of significant correlations observed suggests that ankle adaptations may be influenced by additional factors beyond knee correction magnitude alone, such as individual anatomical variations, soft-tissue constraints, or compensatory mechanisms at other joints. Importantly, hip parameters remained stable regardless of alignment strategy, suggesting that compensations occur predominantly at the distal, rather than the proximal, segment of the kinetic chain.

From a clinical perspective, these findings may support greater attention to ankle alignment and hindfoot/subtalar mobility during preoperative assessment of varus knees, particularly when a substantial coronal correction is anticipated [11,12,22]. Patients with severe varus deformity, pre-existing ankle symptoms, restricted subtalar compensation, or radiographic ankle malalignment may represent a subgroup in whom distal joint response to knee realignment deserves specific consideration. KA may offer a more physiologic approach in such cases by minimising mechanical demands on distal adaptation [23,24].

The strong knee–ankle coupling observed with MA suggests that postoperative ankle symptoms following TKA may represent biomechanical consequences rather than coincidental findings. Preoperative assessment of subtalar range of motion and ankle pathology may help identify patients who would benefit from alignment strategies that preserve constitutional anatomy [23,24]. For patients with severe varus deformity and restricted subtalar mobility, KA may reduce the biomechanical burden imposed on the ankle–hindfoot complex [25].

However, these findings should be interpreted within appropriate boundaries. The absence of clinical outcome data means that biomechanical consequences cannot yet be translated directly into functional or symptomatic predictions.

### Limitations

This retrospective radiographic study lacked patient-reported outcomes and long-term follow-up. Rotational alignment and subtalar kinematics were not assessed.

The non-contemporaneous and multi-institutional nature of the cohorts is an important limitation. MA procedures were performed between 2017 and 2018, whereas KA procedures were performed between 2019 and 2022, reflecting the surgeon’s transition in alignment philosophy. However, all procedures were performed by a single experienced surgeon, using consistent surgical indications which reduces inter-surgeon variability and improves technical consistency.

Prospective studies incorporating clinical outcomes, gait analysis, and subtalar mobility are warranted.

## 5. Conclusions

In patients with varus knee deformity, mechanical alignment (MA) produces larger tibial-driven coronal corrections and increases joint line obliquity (JLO), which induces measurable compensatory valgus shifts at the ground-referenced ankle. Conversely, kinematic alignment (KA) preserves native alignment and JLO, effectively limiting distal radiographic adaptations. This study contributes to the current understanding of knee–ankle biomechanical coupling by demonstrating that the choice of alignment philosophy in total knee arthroplasty extends its influence along the distal kinetic chain. Consequently, careful preoperative assessment of ankle alignment and hindfoot mobility is strongly warranted in patients presenting with severe varus deformity, particularly when substantial coronal correction is anticipated.

## Figures and Tables

**Table 1:** Patient demographics between kinematic alignment (KA) and mechanical alignment (MA) groups, including age (years), sex (number and percentage), weight (kg), height (cm), and body mass index (BMI, kg per m^2^). Values are presented as mean ± standard deviation unless otherwise indicated.

Characteristic	KA (*n* = 32)	MA (*n* = 31)	*p*-Value
Age (years)	71.2 ± 7.2	69.9 ± 8.5	0.531
Sex			0.247
Women	24 (75%)	18 (58.1%)	
Men	8 (25%)	13 (41.94%)	
Weight (kg)	78.9 ± 11.4	78.7 ± 11	0.963
Height (cm)	159.9 ± 7.6	161.3 ± 8.4	0.478
BMI (kg/m^2^)	30.9 ± 4.3	30.2 ± 3.4	0.49

**Table 2:** Baseline radiographic alignment parameters in the kinematic and mechanical alignment groups. Data are presented in degrees. Distribution was assessed with the Shapiro–Wilk test. Normally distributed variables are expressed as mean ± standard deviation and compared with the Welch *t*-test; non-normally distributed variables are expressed as median [interquartile range] and compared with the Mann–Whitney U test. *p* < 0.05 was considered statistically significant. KA, kinematic alignment; MA, mechanical alignment; mHKA, mechanical hip–knee–ankle angle; aHKA, arithmetic hip–knee–ankle angle; MPTA, medial proximal tibial angle; LDFA, lateral distal femoral angle; JLO, joint line obliquity; GTDA, ground–talar dome angle; GTPA, ground–tibial plafond angle; LDTA, lateral distal tibial angle; TCA, talar coverage angle; AJLCA, ankle joint line convergence angle.

Parameter	KA	MA	*p*-Value	Test
mHKA	170.8 ± 3.4	170.8 ± 5.1	0.940	*t*-test
aHKA	−4.3 [−7.8 to −2.3]	−3.9 [−6.7 to −0.7]	0.296	Mann–Whitney
MPTA	85.3 ± 3.0	86.8 ± 4.3	0.135	*t*-test
LDFA	89.5 [88.6 to 92.0]	89.5 [88.8 to 90.9]	0.891	Mann–Whitney
JLO	175.3 ± 4.4	176.9 ± 4.8	0.179	*t*-test
GTDA	90.8 ± 5.6	94.2 ± 6.1	0.025	*t*-test
GTPA	88.9 [84.3 to 92.5]	91.8 [86.3 to 94.7]	0.153	Mann–Whitney
LDTA	84.6 ± 4.7	87.4 ± 4.5	0.021	*t*-test
TCA	11.4 ± 4.6	11.5 ± 3.6	0.893	*t*-test
AJLCA	1.2 [0.6 to 2.4]	0.9 [0.6 to 1.7]	0.509	Mann–Whitney

**Table 3:** Comparison of changes in knee alignment parameters between kinematic alignment (KA) and mechanical alignment (MA). Data presented as mean ± standard deviation for normally distributed variables or median (interquartile range) for non-normally distributed variables. ΔmHKA: change in mechanical hip knee ankle angle. ΔMPTA: change in medial proximal tibial angle. ΔLDFA: change in lateral distal femoral angle. ΔaHKA: change in the arithmetic hip–knee–ankle angle. ΔJLO: change in joint line obliquity. ΔKJLCA: change in knee joint line convergence angle. Effect size reported as Cohen’s d. * Statistically significant difference (*p* < 0.05).

Parameter	KA Change (°)	MA Change (°)	Difference	*p*-Value	Cohen’s d	Effect Size
ΔmHKA	4.82 ± 2.60	8.89 ± 4.99	4.07 (84.4%)	<0.001 *	1.02	Large
ΔMPTA	2.41 ± 1.85	6.26 ± 3.92	3.85 (159.8%)	<0.001 *	1.11	Large
ΔLDFA	−0.70 (−1.97 to 0.58)	−0.70 (−1.90 to 1.80)	0.43 (22.4%)	0.187	0.28	Small
ΔaHKA	0.95 (−1.60 to 2.52)	2.90 (0.30 to 5.30)	3.57 (86.0%)	<0.001 *	0.93	Large
ΔJLO	−0.10 (−2.72 to 1.22)	2.30 (−1.00 to 5.50)	3.80 (126.7%)	<0.001 *	1.92	Large
ΔKJLCA	1.23 ± 1.05	1.41 ± 1.18	0.18 (14.6%)	0.423	0.16	Negligible

**Table 4:** Changes in ankle alignment parameters following kinematic alignment (KA) and mechanical alignment (MA) total knee arthroplasty. Data presented as mean ± standard deviation for normally distributed variables or median (interquartile range) for non-normally distributed variables. Within-group *p*-values from one-sample *t*-tests or Wilcoxon signed-rank tests; between-group *p*-values from independent *t*-tests or Mann–Whitney U tests as appropriate. GTDA: ground talar dome angle. GTPA: ground tibial plafond angle. LDTA: lateral distal tibial angle. TCA: talocrural angle. AJLCA: ankle joint line convergence angle. Asterisks indicate statistically significant differences. * Statistically significant difference (*p* < 0.05).

Parameter	KA Change (°)	*p*-Value	MA Change (°)	*p*-Value	Between Groups *p*	Cohen’s d	Effect
GTDA	−0.85 (−4.67 to 3.17)	0.451	−5.30 (−7.15 to −2.00)	0.006 *	0.012 *	0.91	Large
GTPA	−0.38 ± 2.08	0.478	−3.02 ± 3.91	0.006 *	0.014 *	0.93	Large
LDTA	0.25 ± 1.82	0.614	1.48 ± 2.95	0.058	0.089	0.51	Medium
TCA	−1.35 (−3.42 to 1.65)	0.692	0.40 (−1.15 to 2.30)	0.114	0.156	0.38	Small
AJLCA	−0.10 (−1.50 to 0.82)	0.812	0.20 (−0.75 to 1.20)	0.287	0.412	0.24	Small

## Data Availability

The raw data supporting the conclusions of this article will be made available by the authors on request.

## References

[1] Tian G., Wang L., Liu L., Zhang Y., Zuo L., Li J. (2022). Kinematic alignment versus mechanical alignment in total knee arthroplasty: An up-to-date meta-analysis. J. Orthop. Surg..

[2] Nisar S., Palan J., Rivière C., Emerton M., Pandit H. (2020). Kinematic alignment in total knee arthroplasty. EFORT Open Rev..

[3] Van Essen J., Stevens J., Dowsey M.M., Choong P.F., Babazadeh S. (2023). Kinematic alignment results in clinically similar outcomes to mechanical alignment: Systematic review and meta-analysis. Knee.

[4] Gao Z.-X., Long N.-J., Zhang S.-Y., Yu W., Dai Y.-X., Xiao C. (2020). Comparison of Kinematic Alignment and Mechanical Alignment in Total Knee Arthroplasty: A Meta-analysis of Randomized Controlled Clinical Trials. Orthop. Surg..

[5] Liu B., Feng C., Tu C. (2022). Kinematic alignment versus mechanical alignment in primary total knee arthroplasty: An updated meta-analysis of randomized controlled trials. J. Orthop. Surg. Res..

[6] Nazlıgül A.S., Doğan M., Duran İ., Moya-Angeler J., Akkaya M. (2024). Mid-Term Clinical and Radiological Changes in the Ankle Joint in Varus Knee Osteoarthritis Following Total Knee Arthroplasty. J. Clin. Med..

[7] Shichman I., Ben-Ari E., Sissman E., Oakley C., Schwarzkopf R. (2022). Effect of Total Knee Arthroplasty on Coronal Alignment of the Ankle Joint. J. Arthroplast..

[8] Norton A.A., Callaghan J.J., Amendola A., Phisitkul P., Wongsak S., Liu S.S., Fruehling-Wall C. (2015). Correlation of knee and hindfoot deformities in advanced knee OA: Compensatory hindfoot alignment and where it occurs. Clin. Orthop. Relat. Res..

[9] Cho W.-S., Cho H.-S., Byun S.-E. (2017). Changes in hindfoot alignment after total knee arthroplasty in knee osteoarthritic patients with varus deformity. Knee Surg. Sports Traumatol. Arthrosc..

[10] Diao N., Yu F., Yang B., Ma L., Yin H., Guo A. (2021). Association between changes in hip-knee-ankle angle and hindfoot alignment after total knee arthroplasty for varus knee osteoarthritis. BMC Musculoskelet. Disord..

[11] Jeong B.O., Kim T.Y., Baek J.H., Jung H., Song S.H. (2018). Following the correction of varus deformity of the knee through total knee arthroplasty, significant compensatory changes occur not only at the ankle and subtalar joint, but also at the foot. Knee Surg. Sports Traumatol. Arthrosc..

[12] Choudhury A.K., Bansal S., Pranav J., Raja B.S., Gupta T., Paul S., Gupta K., Bhushan Kalia R. (2024). Increased medial talar tilt may incite ankle pain and predispose ankle osteoarthritis after correction of severity of knee varus deformity among patients undergoing bilateral total knee arthroplasty: A prospective observation. Knee Surg. Relat. Res..

[13] van Drongelen S., Wesseling M., Holder J., Meurer A., Stief F. (2020). Knee Load Distribution in Hip Osteoarthritis Patients After Total Hip Replacement. Front. Bioeng. Biotechnol..

[14] Yun H.H., Lee W., Park J., Choi Y.S. (2024). Change of joint line convergence angle and other coronal alignments after total hip arthroplasty. Orthop. Traumatol. Surg. Res..

[15] Victor J.M.K., Bassens D., Bellemans J., Gürsu S., Dhollander A.A.M., Verdonk P.C.M. (2014). Constitutional varus does not affect joint line orientation in the coronal plane. Clin. Orthop. Relat. Res..

[16] Clark G.W., Steer R.A., Khan R.N., Collopy D.M., Wood D. (2023). Maintaining Joint Line Obliquity Optimizes Outcomes of Functional Alignment in Total Knee Arthroplasty in Patients With Constitutionally Varus Knees. J. Arthroplast..

[17] Zeighami A., Dumas R., Aissaoui R. (2021). Knee loading in OA subjects is correlated to flexion and adduction moments and to contact point locations. Sci. Rep..

[18] Barrios J.A., Higginson J.S., Royer T.D., Davis I.S. (2009). Static and dynamic correlates of the knee adduction moment in healthy knees ranging from normal to varus-aligned. Clin. Biomech..

[19] Howell S.M., Papadopoulos S., Kuznik K.T., Hull M.L. (2013). Accurate alignment and high function after kinematically aligned TKA performed with generic instruments. Knee Surg. Sports Traumatol. Arthrosc..

[20] Nedopil A.J., Howell S.M., Hull M.L. (2020). Kinematically Aligned Total Knee Arthroplasty Using Calipered Measurements, Manual Instruments, and Verification Checks. Personalized Hip and Knee Joint Replacement.

[21] Shichman I., Hallak A., Ashkenazi I., Warschwaski Y., Gold A., Snir N. (2024). Effect of inverse kinematic alignment total knee arthroplasty on coronal alignment of the ankle joint in patients with varus knee deformity. Arch. Orthop. Trauma. Surg..

[22] Xie K., Han X., Jiang X., Ai S., Dai K., Yu Z., Wu H., Qu X., Yan M. (2019). The effect of varus knee deformities on the ankle alignment in patients with knee osteoarthritis. J. Orthop. Surg. Res..

[23] Sangaletti R., Montagna A., Calandra G., Andriollo L., Bna C., Benazzo F., Paolo Rossi S.M. (2025). Robotic functional alignment in knee arthroplasty minimizes impact on ankle alignment: Role of MPTA and LDFA preservation. Knee Surg. Sports Traumatol. Arthrosc..

[24] Franceschetti E., Giurazza G., Donantoni M., Campi S., Tanzilli A., Gregori P., Paciotti M., Zampogna B., Marinozzi A., Longo U.G. (2025). Kinematic alignment, but not mechanical alignment, preserves the knee–ankle relationship after total knee arthroplasty: A retrospective radiographic analysis from the FP-UCBM Knee Study Group. Knee Surg. Sports Traumatol. Arthrosc..

[25] Li Z., de Grave P.W., Van Criekinge T., Luyckx T., Claeys K. (2026). Effect of alignment strategy on lower limb kinematics during stair descent after robot-assisted total knee arthroplasty. Knee Surg. Sports Traumatol. Arthrosc..

